# Multi-Phase Environmental Impact Assessment of Marine Ecological Restoration Project Based on DPSIR-Cloud Model

**DOI:** 10.3390/ijerph192013295

**Published:** 2022-10-15

**Authors:** Junwu Wang, Yipeng Liu, Mingyang Liu, Suikuan Wang, Jiaji Zhang, Han Wu

**Affiliations:** 1 School of Civil Engineering and Architecture, Wuhan University of Technology, Wuhan 430070, China; 2 Hainan Research Institute of Wuhan University of Technology, Sanya 572025, China; 3 China Construction Third Engineering Bureau Group Co., Ltd., Wuhan 430040, China; 4 School of Engineering and Construction, Nanchang University, Nanchang 330031, China

**Keywords:** ecological restoration project (ERP), multi-phase environmental impact assessment, DPSIR, cloud model, Monte Carlo simulation

## Abstract

In order to achieve a comprehensive evaluation of the environmental impact of ecological restoration projects (ERP) under the current destruction and restoration of coastal ecological areas, this paper takes into account the impact of positive and negative indicators on the environment; analyzes the positive and negative benefits of ERP; and establishes a comprehensive environmental impact index system for marine ERP from ecological, economic, and social perspectives through the DPSIR model. On this basis, the cloud model and Monte Carlo simulation are used to obtain the comprehensive assessment grade of the construction period, short-term operation, and long-term operation in the project life cycle. The results show that the benefits of ERP, considering the impact of negative factors, are significantly reduced, and the benefits of ERP will increase remarkably in the long-term operation period. In engineering practice, the environmental pressure factor caused by excessive human activities during construction and operation periods is a key negative factor affecting the overall benefits of ERP. For project decision makers and other stakeholders, the comprehensive assessment grade considering negative impacts is more practical. At the same time, decision makers should take active response measures in the framework of long-term sustainable development, set a tolerance threshold for negative pressure indicators, and strengthen the management of ERP.

## 1. Introduction

After prioritizing ecological conservation and strategies to build China into a strong maritime country, China has continuously strengthened its governance of marine ecological civilization. However, with the rapid development of China’s economy and its industrialization, ecological landscape construction issues crop up frequently in terms of the ecological development, planning, and ecological protection of coastlines. In addition, the construction process of marine engineering, as well as human activities during its use, will also have a direct or indirect impact on the marine environment [1,2].

To effectively address these issues, to promote the construction of marine ecological civilization, and to achieve truly sustainable development, the Chinese government has promoted the implementation of several islands and coastal ERP in recent years, including shoreline control, coastal wetland restoration, and sea area/island construction. According to the “Technical Guide for Marine Ecological Restoration (Trial)” issued by the Ecological Restoration Department of the Ministry of Natural Resources of China in 2021, marine ecological restoration refers to the gradual restoration of the stability of the degraded, damaged, or destroyed natural ecosystems by the use of an ecosystem’s self-restoration capabilities or through appropriate artificial auxiliary measures. These restoration projects can restore the marine topography, optimize the ecological structure, and enhance the ecological function. Furthermore, they can improve the landscape effect of the coastline, and protect and enhance the diversity of regional ecosystems [3,4]. However, coastal resources and environments are often complex and sensitive. ERP are easily damaged by human activities during and after construction, and their self-restoration capacity is poor, which causes coastal ecological restoration areas to face uncertain environmental risks [5]. Therefore, coastal areas must be developed with great care and require appropriate environmental management actions to mitigate impacts. Environmental impact assessment (EIA) analyzes and evaluates the possible impact of a project on the environment through prior scientific investigation [6], which is crucial for the restoration, recovery, and protection of the ecological environment and forms the basis of many management practices [7].

However, the current research is limited to the positive evaluation of the ecological restoration effect, as well as the evaluation of the ecological, social, and economic benefits after the implementation of the project [8], [9] in order to maximize such multi-level benefits. It is still difficult to comprehensively evaluate the negative impact of the environmental destruction caused by the process of constructing ERP, the favorable influence of ecological restoration as a result of the completion of the project, and the uncertain influence of human activities on the marine ecological environment after the completion of the project restoration. The ecological and social benefits of the project cannot be accurately positioned by conducting the EIA of the project construction or the project operation period. The ERP should take into account the environmental impact in the construction process, as well as the short-term and long-term environmental impact after construction.

In this paper, comprehensive evaluation of the short-term construction and long-term operation of the ERP is achieved by comprehensively considering the environmental impact factors of the whole process of ERP construction and operation. The evaluation results are extremely important for the long-term sustainable development of marine resources and the environment.

## 2. Literature Review

### 2.1. Research Status of Coastal Ecological Restoration Evaluation

Many scholars have carried out various studies on ecological restoration assessment. However, most studies have focused on comparative analysis after the completion of targeted projects, putting forward measures and technologies for constructing ERP, and evaluating the technologies and schemes from the perspectives of marine health and other aspects [10,11,12]. However, the impact of these projects on the sensitive coastal environment is not mentioned, and human activities also have a destructive impact on the restored coastal environment. Domestic and foreign research both still lack a comprehensive consideration of the restoration process.

Some scholars analyzed the results of the ERP within a short timeframe (within 5 years) after its construction [13,14,15], and evaluated the ERP from the perspectives of the degree of human intervention and the motivation of restoration through information evaluation. It is pointed out that a lack of community management is one of the reasons for the failure of ecological modification projects; the effect of restoration depends on the relevant policies, the surrounding social, economic and political environment, and the interests of stakeholder groups. Ounanian also pointed out that many coastal ERP are only providing short-term results of 1–2 years, and long-term (15–20 years) results-based evaluation still needs to be strengthened. This is consistent with the conclusion of [16]. For ERP, short-term analysis and evaluation are not enough, and evaluating project value from the perspective of a single benefit is also limited. Public participation, community supervision, and management may be a means to enhance marine restoration projects.

The restoration of the marine ecosystem faces different forms of uncertainty, such as incomplete knowledge, model uncertainty, and decision-making uncertainty. A good way to reduce these uncertainties is by coping with the incomplete knowledge in the model and by simulating the decision-making process through many random numbers.

### 2.2. Research Status of Evaluation Methods

Ecological assessments are essential for the restoration, recovery, and protection of ecological environments and form the basis for many management practices. Chen analyzed and evaluated three marine ecosystem restoration cases in China and pointed out that most of the schemes focused on restoration measures but lacked analysis of the causes of ecosystem degradation as well as the assessment [17], evaluation, and management research of projects. Regarding the use of evaluation methods, the current EIA of the ecological restoration system is mainly based on qualitative analysis, and the ecological and economic effects of the project are evaluated through case analysis [18,19]. Compared with impact assessment based on complexity theory [20,21] and the theory of risk society [22], environmental social impact assessment (ESIA) [23] and PSR (Pressure, State, Response) [24,25] models, which are widely used in traditional project environmental assessment and planning environmental assessment, are not developed enough due to the lack of studies on ecological restoration effect evaluation and restoration management in ERP.

The DPSIR (Driving, Pressure, State, Impact, Response) model improved by the European Environment Agency (EEA) [26] based on PSR is also widely used in the study of large marine ecosystem health [27], coastal and marine fishery resource management [28], the drivers of coastal environmental degradation [29], and marine management, integrating ecosystem services and social benefits [30].

DPSIR can not only characterize the impact of social activities and project construction on the marine ecological environment, and the feedback of environmental status on society but also reflect the social policy response involved in maintaining the sustainable development of the environmental system [31,32]. It is conducive to a more complete analysis of the social, ecological, and economic factors to involve the decision makers and stakeholders of the complex marine environmental system [33]. Moreover, in ERP, people participate in the transformation of and improvement in the damaged coastal ecosystem and pay more attention to the social and environmental impact of the project. Therefore, DPSIR is a highly suitable method for the evaluation of marine ERP [27].

Currently, there is a lack of comprehensive consideration of EIA in the marine ecological environment during the construction and operation periods, and a lack of scientific and systematic analysis of influencing factors in the comparative analysis of examples that are mainly used in environmental assessment methods. The complexity of marine ERP demands that the comprehensive impact assessment of the project must include the whole process of the construction and operation periods, and center on quantitative analysis, i.e., multiple assessment indicators, multi-objective decision-making methods, or models adopted from a multiple-phase perspective. In addition, unlike the process of regional ecosystem environmental assessment, most or all of the evaluation indicators are quantitative indicators of the region’s previous economy, environment, and society [34,35,36], such as population and GDP. The environmental assessment process of ERP refers to an evaluation of the project construction and possible future development. It is a long-term process for the future, and it is difficult to use quantitative regional indicators to accurately measure the impact of a project on the environment. Therefore, many initial indicators for ERP are qualitative descriptions, which need to be transformed into quantitative evaluations through fuzzy mathematics. The cloud model is based on fuzzy mathematics and stochastic mathematics, which can convert stochastic and fuzzy qualitative evaluation indicators into quantitative indicators [37]. This superior solution has strong quantitative and qualitative analysis characteristics [38]. Furthermore, it has been widely used in evaluation and analysis in various fields, such as chemical industry safety evaluation [39], seabed risk assessment [40], and ecological environment assessment [37,41], etc. The cloud model is suitable for comprehensive evaluation when dealing with complex uncertain problems and for the comprehensive evaluation of the ecological environment.

To sum up, in this paper, the DPSIR-cloud model method is used to analyze and evaluate the ecological and environmental impacts caused by human, social, economic, and other activities in the construction and operation of ecological restoration and to comprehensively consider the positive and negative environmental impacts during the implementation and operation of the project. The EIA framework of the marine ERP is constructed, and Monte Carlo simulation is used to reduce the uncertainty of the comprehensive assessment for the phase that is more difficult to quantify in the evaluation process.

## 3. Materials and Methods

The DPSIR model, proposed by the European Environment Agency(EEA), is a multi-level theoretical model for scientific planning and evaluation in environmental systems [42], which can clearly and concisely reflect the causal relationship and mutual feedback effects among economic, social, resource, environmental, and policy indicators by constructing an evaluation index system based on five dimensions. It can check the effects of an impact among the environment, economy, and society from the perspective of system analysis to provide a practical and rational theoretical basis for stakeholders’ decision making.

### 3.1. Index System of Ecological Restoration Evaluation Based on DPSIR

In this paper, the DPSIR model framework is used to construct the index system for the ERP. Based on the relevant literature and the actual characteristics of ERP, the coastal ecological security evaluation index system began from the environmental impact of coastal ecological projects and the connotation of the effectiveness of ERP and was constructed by the use of the top-down and layer-by-layer decomposition methods, with a total of 15 indexes. These indicators were sourced from “Technical Specifications for Ecological Environment Status Evaluation HJ 192-2015”, “Feasibility Study Report of Project”, and some previous documents [3,19,43,44,45,46] and are divided into five cyclic subsystems, as shown in Table 1: driving (D), pressure (P), state (S), impact (I), and response (R) [32].

Driving (D): The social and economic activities that have a potential impact on regional environmental changes are the imminent causes of ecological environment changes and future trends. The government promotes the ERP to improve the regional ecological environment and the quality of life of the surrounding residents and to restore the coastline. Social development (D1) and demographic factor (D2) are the main measurement factors.

Pressure (P): the pressure and direct impact of social development and human activities on the ecological environment in the region. It mainly includes construction land, development intensity, and human activity intensity. The objective of the ERP is to improve the coastal environment, so the project has a positive influence on the environment in general, but it also has a negative influence because the implementation of the project will inevitably destroy or pollute the original environment. As the regional climate conditions are close to the ocean, the natural environment is unique, complex, and sensitive. In the case of human disturbance, restoration takes a long time. The most pressing factors include the construction pollution intensity (P1), the damage intensity (P2) to the original environment, and the pollution intensity (P3) brought about by the operation period.

State (S): This refers to the various conditions presented by the ecological environment affected by the driving force and pressure in the region, including the expected ecological restoration (S1), the improvement (S2) of tourism resources, and the pollution (S3) caused by human activities during operation after the project is completed.

Impact (I): The response and impact of the ecological environment system on the economy, society, and resources of a region provide a reference for government decision making. This mainly includes the direct economic benefits brought about by the improvement of the environment and the economic benefits (I1) of land appreciation. The completion of the project increases the forest coverage rate, improves the polluted ecological environment, leads to the expansion of biodiversity and other environmental benefits (I2), enhances the overall development level of the city, and improves the social benefits (I3) through the ecological restoration of the coastline, the repair of the landscape repair, and the improvement of facilities.

Response (R): The corresponding solutions are made by the government according to the feedback in terms of resource consumption, ecological environment state, and impact index information. For ERP, the primary factor is the government investment in the project (R1), the second is the degree of control over pollution (R2) during the construction process, and the last is the participation and management level (R3) in the operation period after the project is completed.

The driving factors include both economic development (D1), which is favorable to ERP, and negative factors (D2) brought about by rapid population growth. The driving factors bring about the construction of the project, as well as environmental pressure (P), including the negative impact during operation and construction. The common impact of the driving factors and environmental pressure produces the ERP and its surrounding states (S), thus bringing about a series of ecological, social, and economic benefits. Managers need to actively respond to the above-mentioned changes and take corresponding measures to strengthen management. The above-mentioned index impact process is a simplified simulation of the entire ERP system, and the comprehensive evaluation of the environment also needs to analyze the index attributes.

The ERP aims to bring the regional ecosystem to its optimal state and to achieve the expected goal, so the goal of restoration must be to provide the best functional result [47] and to achieve the expected ecological restoration benefit. This benefit is a complex of ecology, society, and economy [48]. Generally, in a comprehensive evaluation, the range standardization method is used to deal with positive and negative indicators during comprehensive evaluation [49]. However, in order to unify the standard of comparison and to ensure the reliability of the results, we need to deal with the original variables before analyzing the data to eliminate the influence of different attributes between different indicators, so that the results are more comparable.

Assuming that *n* indexes are given, such as $X_{1},X_{2},\cdots ,X_{n}$, including the positive indexes $X_{i}^{+}\in \left\{X_{1}\right.,X_{2},\cdots ,\left.X_{k}\right\}$ and negative indexes $X_{i}^{-}\in \left\{X_{1}\right.,X_{2}\cdots ,\left.X_{n-k}\right\}$, the index score is $x_{i}=\{x_{1},x_{2},\cdots ,x_{n}\}$. Generally, assuming that the standardized values for every indicator data are $Y_{1},Y_{2},\cdots ,Y_{k}$, then

Standardization of positive index

(1) $$Y_{ij}=\frac{x_{ij}-\min(x_{i})}{\max(x_{i})-\min(x_{i})}$$

Standardization of negative index

(2) $$Y_{ij}=\frac{\max(x_{i})-x_{ij}}{\max(x_{i})-\min(x_{i})}$$

However, the normalization of the data usually converts all indexes into positive ones. Although negative indexes are also considered at this time, the comprehensive evaluation of positive indexes is carried out in essence. In Formulas (1) and (2), *Y_ij_* > 0, weight $w_{i}\in (0,1)$, and comprehensive evaluation $\forall x_{i}\in \left\{x_{1}\right.,x_{2}\cdots ,\left.x_{n}\right\}$, $E=\sum_{i}^{n}w_{i}x_{i}>0$.

For decision makers, the result of positive evaluation reflects that they pay more attention to the indexes with high positive scores and may ignore the impact of negative indexes. However, the comprehensive assessment of the environmental impact of ERP needs to be analyzed in terms of positive and negative benefits. From the perspective of comprehensive environmental impact, negative indexes are indicators of environmental damage. Decision makers should pay attention to the benefits brought about by positive indexes, and those extreme indicators that cause great damage to the environment. Negative indexes should not be converted into positive ones for evaluation.

For decision makers, negative indexes reduce the comprehensive environmental benefits of the project during a comprehensive evaluation of ERP. If the negative impact is too large, the overall benefits may be negative, so the weight of negative indexes is negative,$w_{X_{i}^{-}}\in (-1,0)$; the greater the index score, the greater the impact, and all indexes should be in accordance with Formula (1) $\exists x_{i}\in \left\{x_{1}\right.,x_{2}\cdots ,\left.x_{n}\right\}<w_{i}\in \left(-1,0\right)$ so that the comprehensive evaluation result is $E=\sum_{i}^{n}w_{i}x_{i}<0$. This shows the existence of some negative factors which will make the benefits of ERP negative if their influence is too large. When using the cloud model for a comprehensive evaluation, the cloud parameters of negative indexes are also negative as these indexes reduce the comprehensive evaluation of the project by the stakeholders.

The neutral index represents that its impact on the environment can be positive or negative. For example, index R3, low participation, and management level in the operation period lead to disordered management and pollution and destruction activities, resulting in a negative impact on the environment. The success of the ERP is closely related to the management in the operation period. The environmental impact of the ERP is determined according to the evaluation score. The median value is 0.5. An average score above 0.5 indicates a positive impact on the environment, while an average score below 0.5 indicates a negative impact on the environment.

### 3.2. Environmental Impact Assessment Based on the Cloud Model

Definition: Assuming that *U* is a quantitative domain, *C* is a qualitative concept in the quantitative domain *U*; the quantitative value *x* is a stochastic realization of *C*, i.e., ∈x∈U; and the random number *μ*(*x*) is the degree of certainty that *x* realizes on *C*, $\mu (x)\in \left[0,1\right]$, and reflects the degree of certainty of the quantitative value *x* to *C*.

Cloud droplets are the elements of clouds. According to the data and the normal cloud generator, the model parameters *C* (*Ex*, *En*, and *He*) are selected. *Ex* is the expectation of the distribution of cloud droplets in *U*, the entropy *En* is the uncertainty of *C*, and the hyper entropy *He* is the uncertainty of *En*, i.e., *En* reflects the dispersion degree of cloud droplets [45].

**Figure 1:**
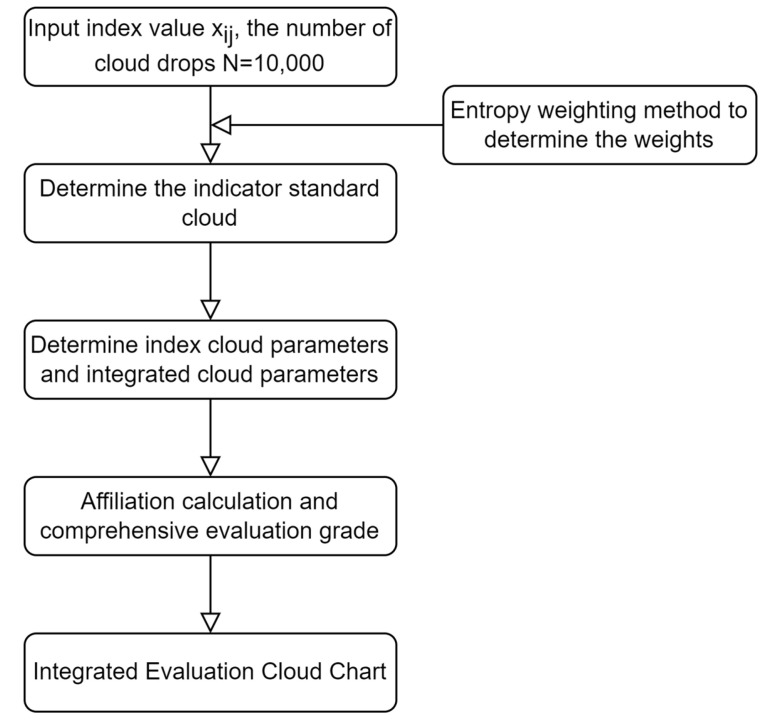
Evaluation process of the cloud model for the ERP.

The ERP evaluation model based on the cloud model is shown in Figure 1.

The detailed steps are as follows:

(1) Determination of comment sets based on DPSIR model indexes

According to China’s National Environmental Protection Standards (Technical Specifications for Ecological Environment Assessment), the ecological environment state is divided into five grades, namely excellent, good, medium, inferior, and poor. Based on the DPSIR model, this paper classifies the ERP evaluation index into Grade I (small), Grade II (little), Grade III (medium), Grade IV (relatively great), and Grade V (great).

(2) Determination of index weight

In this paper, the entropy weight method is used to calculate the index weight. It is frequently used in the fields of multi-objective decision making, in comprehensive evaluation, and in studies combined with methods such as the cloud model TOPSIS [49,50,51].

Assuming that the initial data index matrix is $R=\left[x_{ij}\right]$, where *i* = 1, ……, *m*, *j* = 1, …, *n*, *m* represents the number of evaluation objects, and *n* indicates the number of evaluation indexes. The detailed steps to determine the weight are as follows:

 1) Data standardization

The index is processed according to Formula (1).

 2) Information entropy of each index

The information entropy of a set of data is shown as Equation (3):

(3) $$E_{j}=-{\left(\ln n\right)}^{-1}\sum_{i=1}^{n}p_{ij}\ln p_{ij}$$

where $p_{ij}=Y_{ij}/\sum_{i=1}^{n}Y_{ij}$, if $p_{ij}$=0, then $\underset{p_{ij}=0}{\lim}p_{ij}\ln p_{ij}=0$.

 3) Determination of each index weight

The weight of each index by information entropy is calculated by Equation (4):

(4) $$w_{j}=\frac{1-E_{j}}{k-\sum E_{j}}(j=1,2,\cdots ,k)$$

(3) Determination of the cloud eigenvalue of the corresponding grade for each index

 1) Standard cloud parameters

The grade determined in step (1) is quantified as the interval (*C*_min_, *C*_max_) of the cloud model (0, 1), and the corresponding interval grades are Grade I (0, 0.2), Grade II (0.2, 0.4), Grade III (0.4, 0.6), Grade IV (0.6, 0.8), and Grade V (0.8, 1.0). Standard cloud parameters for each index are calculated according to Equation (5), where k is a constant, usually 0.01 [52,53]. The corresponding standard cloud parameters are shown in Table 2.

(5) $$\left\{\begin{array}{l} Ex=\left(C_{\max}+C_{\min}\right)/2\\ En=\left(C_{\max}-C_{\min}\right)/6\\ He=k \end{array}\right.$$

 2) Calculation of cloud model parameters for each index

According to the practical data, the expectation *Ex* and variance *S*_2_ are first calculated, and then, entropy *En* based on the sample mean *Ex* and, finally, the hyper entropy *He* are obtained. The specific calculation process is shown as Equation (6).

(6) $$\left\{\begin{array}{l} Ex=\frac{1}{n}\sum_{i=1}^{n}x_{i}\\ S^{2}=\frac{1}{n-1}\sum_{i=1}^{n}{\left(x_{i}-Ex\right)}^{2}\\ En=\sqrt{\frac{\pi }{2}}\frac{1}{n}\sum_{i=1}^{n}\left|x_{i}-Ex\right|\\ He=\sqrt{\left|S^{2}-En^{2}\right|} \end{array}\right.$$

 3) Construction of a cloud model map for each evaluation index belonging to each risk level

Firstly, the standard cloud map is calculated and then drawn on the basis of the actual model parameters and the realization algorithm of a normal cloud generator to supplement cloud droplets, obtaining the membership of each index corresponding to each grade. Firstly, the normal random number with *En* as the expected value and *He* as the variance is established. Secondly, the normal random number *x*(*i*) with Ex as the expected value and *En*^′^ as the variance is generated. Thirdly, the certainty degree *y* is calculated, i.e., the membership *μ*, and finally (*x*, *y*) is made as a cloud droplet in the domain, as shown in Equation (7).

(7) $$\left\{\begin{array}{l} E_{n}{}^{\prime }=randn(1)\cdot He+E_{n}\\ x(i)=randn(1)\cdot En_{n}+Ex\\ y(i)=\mu =e^{-\frac{{\left(x-Ex\right)}^{\;\;\;2}}{2{\left(En\right)}^{\;\;2}}} \end{array}\right.$$

**Figure 2:**
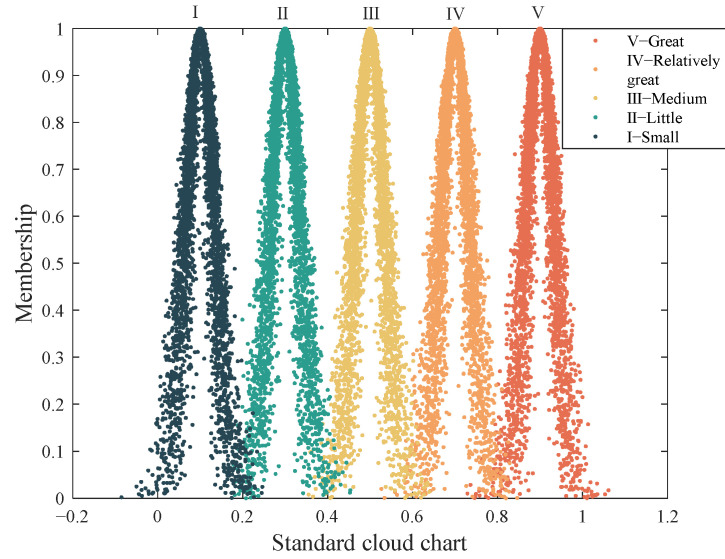
Standard cloud map of the evaluation interval.

According to the standard cloud parameters, the standard cloud map is drawn using Matlab, as shown in Figure 2. According to the determined cloud model parameters, the evaluation cloud is drawn using Matlab software according to Equation (7).

 4) Membership calculation and comprehensive risk assessment level

According to Equation (8), the weight determined by the cloud model above is combined with the cloud parameters of each index to obtain the cloud parameters of comprehensive construction safety risk:

(8) $$\left\{\begin{array}{l} Ex=\sum_{i=1}^{n}Ex_{i}w_{i}\\ En=\sqrt{\sum_{i=1}^{n}{\left(En_{i}w_{i}\right)}^{2}}\\ He=\sqrt{\sum_{i=1}^{n}{\left(He_{i}w_{i}\right)}^{2}} \end{array}\right.$$

where *Ex_i_*, *En_i_*, and *He_i_* refer to the cloud expectation, entropy, and hyper entropy of each index, and *w_i_* is the weight of each index.

The final evaluation integrated cloud of the ERP is obtained, and the cloud map is drawn according to Equation (9). For the membership *μ* of each standard cloud, the final corresponding risk level is determined as the EIA grade of the marine ERP according to the principle of maximum membership and the similarity of the cloud map:

(9) $$\mu_{j}=e^{-\frac{{\left(Ex-Ex_{j}\right)}^{\;\;\;2}}{2{\left(En_{j}\right)}^{\;\;\;2}}}$$

where 0 ≤ $\mu_{j}$ ≤ 1, with $\mu_{j}$ representing the membership; *Ex* is the comprehensive cloud expectation; and *Ex_j_* and *En_j_* are the standard cloud expectation and entropy of each grade, respectively.

Monte Carlo simulation, proposed by IPCC as an effective method to deal with uncertainty, is also a feasible method to predict future development [54,55,56]. Therefore, the Monte Carlo simulation of expert scoring can reduce the uncertainty and fuzziness to some extent. On this basis, this paper uses Monte Carlo to simulate expert scoring in order to predict the future environmental state of SOP during the construction and operation of the project.

**Hypothesis** **1** **(H1):** *The change in index grade between the operation period and the construction period is within a certain range, i.e., the change boundary of the index grade is between the operation period and the construction period. Assuming that experts in each phase of the index score relationship is* $D_{CP}\in \left[D_{SOP},D_{LOP}\right]$.

**Hypothesis** **2** **(H2):** *The scoring probability of each expert on the index at each grade is the same, i.e., the score of each index follows a uniform distribution of* $\left[D_{SOP},D_{LOP}\right]$.

In the next section, the cloud model evaluation method will be used to analyze the real case.

## 4. Results

### 4.1. Case Introduction

The modeling approach constructed in this paper is applied to a beach ERP in Yazhou District, Sanya City, Hainan Province. The project is located along the coastline from No. 7 Road to the outlet of Ningyuan River in Yazhou Bay Technology City, Sanya. The first phase of the project has a planned land area of approximately 164,000 m^2^, a total project length of approximately 1.26 km, and an average width of approximately 130 m. The construction of the project is financed by government investment. The purpose of the project is to ensure that the artificial shoreline remediation and restoration meet the ecological shoreline standard, increase the retention of natural shoreline resources, significantly improve the ecological carrier conditions of the shoreline, optimize the ecological structure, enhance the ecological function, and improve the landscape effect of the shoreline through the construction of the project.

### 4.2. Model Generation

The invited experts came from the construction industry (both constructors and owners), universities, and research institutions. Two experts from each unit, a total of 10 experts, were not invited to judge the public because of the prospective nature of the evaluation content, including the construction and operation periods, and the relatively professional content. The specific information of the experts is shown in Table 3. Indexes of the ERP during the construction and operation periods were scored according to the EIA index, and the expert scores were collected. According to the cloud parameter calculation formula, the cloud parameters of the probability cloud model and the consequence cloud model of each index were calculated.

#### 4.2.1. Cloud Parameter Calculation in the Construction Period (CP)

The cloud parameters of each index in the construction period and the integrated cloud parameters of environmental assessment for ERP were calculated by Formulas (6), (7), and (8), as shown in Table 4.

**Figure 3:**
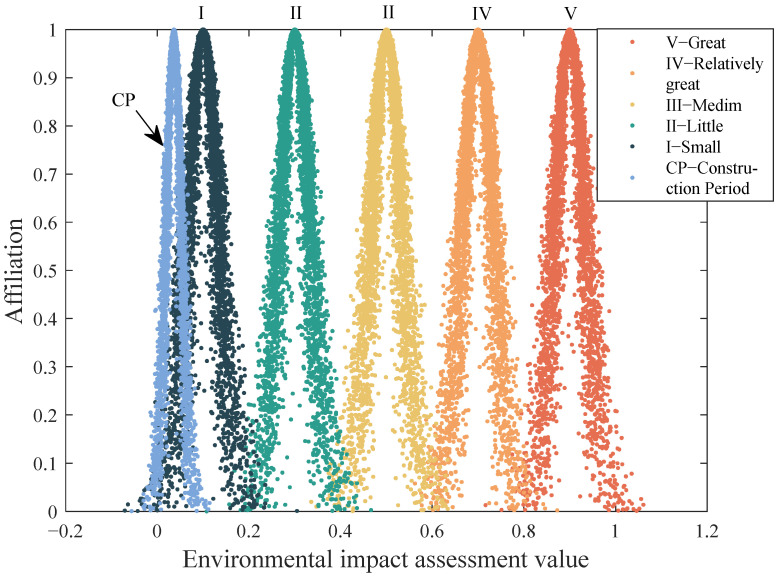
Cloud map for a comprehensive evaluation in the construction period.

The weight was determined by the entropy weight method, and the comprehensive EIA cloud parameters (*Ex*, *En*, *He*) = (0.0362, 0.0167, 0.0044) were calculated by Formula (8). The membership of each risk level (μ1, μ2, μ3, μ4, μ5) = (0.1601, 0, 0, 0, 0) was calculated on the basis of the standard cloud parameters according to Formula (9), since only four valid digits were retained for the data in this paper. The cloud model affiliation degrees μ2, μ3, μ4 and μ5 were approximately equal to 0 in the CP, the calculation of the cloud parameters in the LOP and SOP was the same as in the CP, and the cloud model map for comprehensive evaluation was drawn by the use of MATLAB software, as shown in Figure 3.

According to the calculation results of the standard cloud parameters and the cloud model parameters of each index in Table 2, the greater the expected value in the standard cloud model, the greater the impact. The expected values of P1 and P2 are the largest when the tunneling parameters are not adjusted in time. The expected values of the I2 and R1 indicators are slightly smaller than those of P1 and P2, which indicates that the negative indexes P1 and P2 have a greater negative impact on the environment in the construction process. Pollution and damage to the original environment in the construction period require attention, but in general, the impact on the environment in the construction period is positive and small.

#### 4.2.2. Cloud Parameter Calculation in the Long-Term Operation Period (LOP)

Assuming that 5–10 years after the completion of the project constitutes a long-term operation period, the parameters of the index cloud model are also calculated according to the expert rating level, as shown in Table 5.

**Figure 4:**
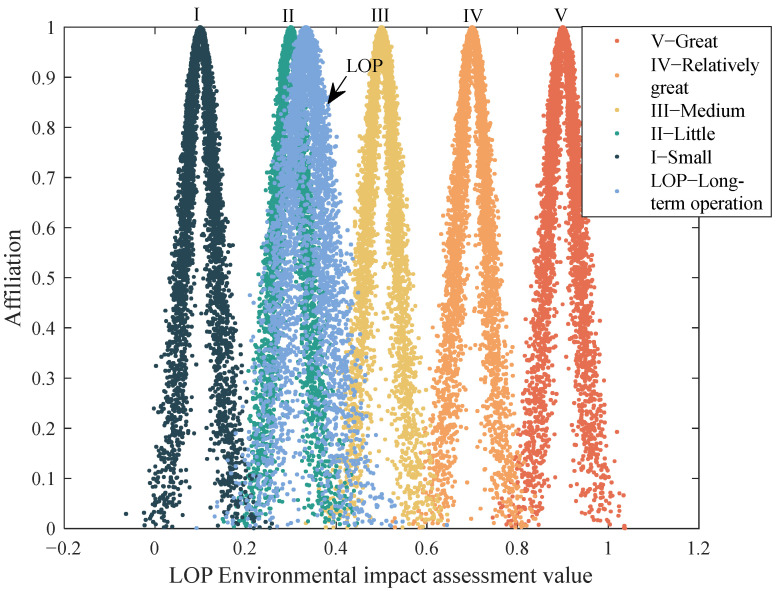
Cloud map for a comprehensive evaluation in the long-term operation period.

Then, the weight of each index in the operation period is calculated by the entropy weight method, and the cloud parameters (*Ex*, *En*, and *He*) of the comprehensive EIA in the operation period are calculated by Formula (8). The affiliation (μ1, μ2, μ3, μ4, μ5) = (0.000, 0.6090, 0, 0, 0) of the index grade is calculated by the standard cloud parameter according to Formula (9), and the cloud model map for a comprehensive evaluation in the construction period is drawn using Matlab software, as shown in Figure 4.

Among the indexes, I2 has the highest expectation and belongs to the highest grade, indicating that experts agree that under the background of China’s promotion of ecological civilization construction, Sanya, Hainan Province, is a pilot city for urban restoration and ecological restoration (double restoration). The driving force (D) allows the project to run smoothly. After the completion of the project, the expected ecological environment improvement effect will be achieved, and the environmental benefits will be outstanding. It is noteworthy that the comprehensive score of the index R3 is lower than 0.5, and its weight is negative in this paper, indicating that experts believe that in LOP, the response R to project management will be insufficient, and they doubt the effect of community participation and management level in the long-term operation period. The participation of stakeholders mentioned in the International Principles and Standards of Ecological Restoration Practice is extremely important to achieving the ecological restoration goal because it can provide policy and financial support for the long-term sustainable development of the project and relieve the possible contradictions [57]. In the long run, the low level of participation and management obviously cannot play a positive role and will therefore have a negative impact on the long-term sustainable development of the project.

#### 4.2.3. Monte Carlo Calculation of Cloud Parameters in the Short-Term Operation Period (SOP)

**Figure 5:**
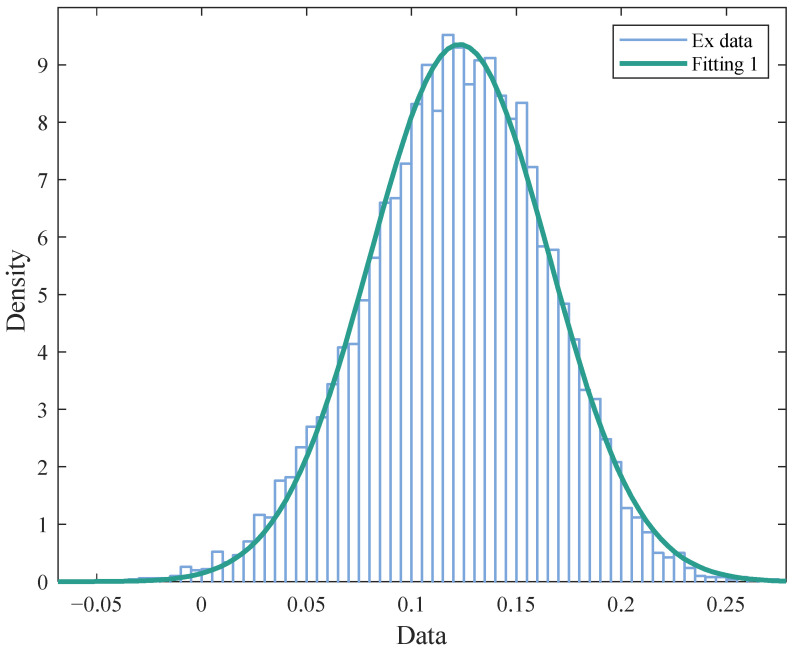
Cloud parameter simulation diagram in SOP.

The scoring of each index in SOP is simulated in Matlab. According to the construction process of Model 3.2, the comprehensive cloud parameters are calculated by the entropy weight method and the cloud model, and the simulation number *n* = 10,000 is set. The Monte Carlo simulation results in the SOP are shown in Figure 5.

The integrated cloud expectation of environmental impact in SOP is consistent with a normal distribution. The interval of the mean distribution is estimated to be (0.1229, 0.1235) under the 95 % confidence interval. According to the membership function of the cloud model, the membership (μ1, μ2, μ3, μ4, μ5) = (0.7898, 0, 0, 0, 0) of the comprehensive evaluation of environmental impact in SOP is calculated, and the overall grade is found to be low. This shows that the overall environmental benefit of the ERP is positive but not high in the short-term period after the completion of the project.

## 5. Discussion

In general, the environmental assessment of the project is based on certain expert judgments, and the qualitative indexes are transformed into quantitative indexes. Although the cloud model can deal with the fuzzy randomness of index transformation, it cannot solve the uncertainty of expert judgment. The construction period and long-term operation period tend to be relatively stable periods, which is convenient for experts to evaluate the grades on the basis of the indexes. Therefore, the uncertainty of cloud parameters is low. Moreover, the direct process from the construction period to the long-term operation period is more likely to be a black-box system and, therefore, difficult to measure. The state between the construction period and the long-term operation period of the project cannot be accurately distinguished, and the uncertainty and fuzzy randomness are also higher than those in the other two phases. [58] pointed out that it is basically impossible to predict the future development of environmental projects with low uncertainty, but one can try to understand a possible future development scope through scenario analysis or other reasonable methods.

**Figure 6:**
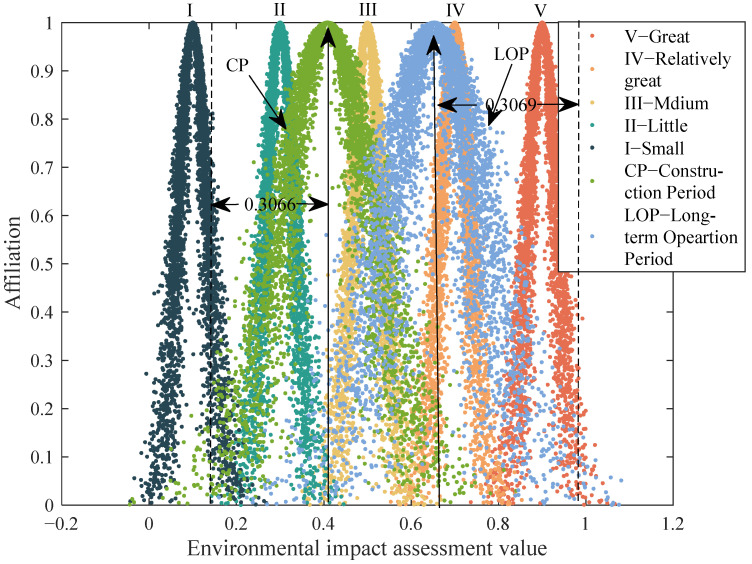
Cloud map of comprehensive evaluation in the construction period (CP) and long-term operation period (LOP).

**Figure 7:**
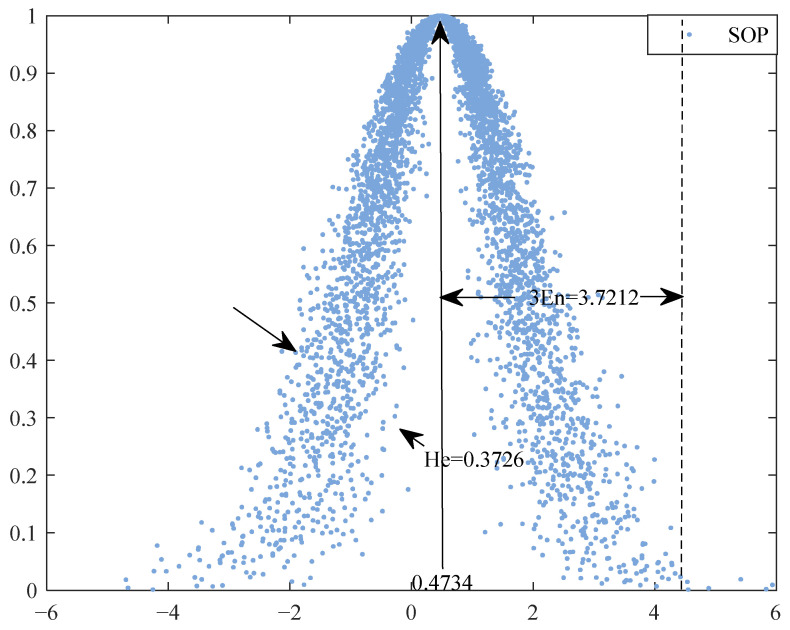
Cloud map for comprehensive evaluation in the short-term operation period (SOP).

The cloud parameters of the ERP in the three phases are calculated through the cloud model and Monte Carlo simulation without considering the negative impact. *En* and *He* are relatively large in the cloud parameters in the short-term operation period, so they are not suitable to be placed in one picture with the cloud map in CP and LOP. The comprehensive grade of environmental impact in the three phases and the cloud map are shown in Figure 6 and Figure 7.

From the perspective of cloud droplet dispersion and cloud thickness in the cloud image, the uncertainty and fuzziness in the EIA are higher without considering the influence of negative factors. This is because the interval of the Monte Carlo simulation data hypothesis is sourced from expert evaluation, and the uncertainty and randomness are great. However, compared with the uncertainty of the short-term operation period, which is difficult to judge by experts, the results are still acceptable.

**Figure 8:**
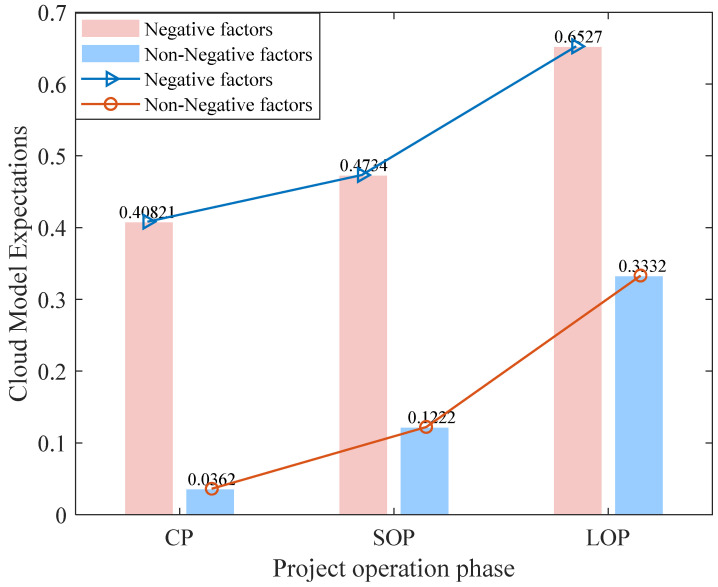
Comprehensive assessment results of environmental impact in three phases of the ERP.

The comprehensive evaluation results of the environmental impact of ERP during the construction period, short-term operation period, and long-term operation period are shown in Figure 8.

It is shown that the comprehensive evaluation of each phase in the construction period and short-term operation period is positive, but the comprehensive benefit is not high, reaching Grade I (low). However, the comprehensive benefit in the long-term operation period increases significantly, reaching Grade III (medium). After considering the impact of various negative indexes, the positive environmental impact of ERP is not as large as expected. The evaluation results of ERP in the construction period without considering the impact of negative factors are basically consistent with the environmental impact evaluation results of projects prepared by the construction party.

However, the environmental evaluation prepared by the construction party pays less attention to the actual impact of the project operation and the effectiveness of mitigation measures. The comprehensive evaluation of projects in the operation period is affected by both positive and negative factors. From the perspective of the positive benefits of ecological restoration, it has economic, social, and ecological benefits at any phase. However, for decision makers and the public, it is not advisable to implement relevant decisions only due to the positive factors, as the negative factors can affect the expected benefits of the project. Decision makers cannot only rely on the EIA report prepared by the construction party when making decisions with regard to ERP. After comprehensively considering the positive and negative factors, the positive benefits in the construction period and the short-term operation period are not great, and the comprehensive benefits can be significantly increased after long-term operation. The negative factors of the environment will lead to a decline in the overall comprehensive benefits at any phase, especially in the construction period. This stage is of the most concern to current decision makers. The purpose of a comprehensive evaluation is to discover the negative effects of negative factors on the overall project, rather than to tell decision makers and stakeholders that the expected returns of the project are not as high as expected or the project is not feasible. Different stakeholders have different purposes in the project construction process, with each party pursuing different benefits, and the government needs to take social responsibility for regional economic and environmental development [59]; hence, it is necessary to provide relevant suggestions for decision makers from their own perspective. Policy makers should strengthen the management of negative factors, suppress negative influencing factors, and enhance positive influencing factors.

In addition, the ecosystem itself has a certain restoration capacity, but the speed of destruction is usually greater than that of restoration. For ERP, it is difficult to accurately estimate the threshold of unacceptable damage. Therefore, it is necessary to set a larger protective safety margin for managers, i.e., to set a lower threshold for negative factors, which can not only improve the overall efficiency but also prevent irreversible damage to the ecological environment caused by negative factors.

Finally, one of the objectives of ecological restoration and sustainable development, as mentioned by [43], is to balance short-term needs against long-term needs and to avoid damaging long-term benefits for the sake of short-term benefits. From the perspective of the comprehensive evaluation of ecological restoration, the benefits of the construction period and the short-term operation period cannot meet the needs of stakeholders after considering the impact of negative factors, but the long-term benefits can. The environmental impact of ecological restoration projects must be judged within the framework of sustainable development. Decision makers and managers need to formulate long-term all-around sustainable development plans and implement the long-term management of ERP.

We give two suggestions based on the above analysis.

(1) Dynamic monitoring technologies, such as remote sensing, GIS systems, and GPS, can be used together with continuous monitoring during pre-restoration, restoration, and post-restoration to more accurately quantify the impact of each index and to provide a more scientific and effective basis for managers and stakeholders to make decisions, which is conducive to the sustainability of marine ecological restoration.

(2) In addition to relying on local financial support, the sources of funding for restoration work also need to guide the broad participation of industrial investment and financing service platforms, commercial financial institutions, and social capital; to strengthen the ecological compensation mechanism; and to improve the management of the use of funds. Project supervision shall focus on the views of stakeholders and professionals, as well as the public, supporting public opinions and social supervision during the whole process of project construction and operation.

The comprehensive evaluation model proposed in this paper includes three stages: the construction period, short-term operation period, and long-term operation period. At present, this project is under construction, and the quantitative data of relevant indicators need to be determined based on the comprehensive results of the three stages, especially the relevant data in the project operation period. Therefore, to achieve the expected comprehensive evaluation in the long-term state, relevant indicators are rated by the expert evaluation method in this paper, which makes the analysis of the proposed model subjective to some extent. In view of this deficiency, the quantitative data expected to be acquired in the future are analyzed, with a view of providing a certain reference for the research in the future.

For driving indicator D, this region is an advanced base of the seed industry and deep-sea research in China, and a large number of domestic and foreign researchers will settle there in the future. Based on the amusement demand for coastal eco-parks, the rapid economic development in this region can provide good support for the construction of this project. Therefore, an analysis can be made from the perspective of regional GDP and population size.

In terms of the pressure indicator P, first of all, as this region is an estuary, mountain torrents happen frequently in typhoon periods due to long-term improper management of the original environment, sewage discharge, and the occupation of a watercourse, thereby causing great damage to the environment. Secondly, it is planned to demolish a certain number of facilities and clear the land again during project construction, which will destroy the existing environment to a certain extent. Moreover, after the park is developed, human activities will also destroy the existing vegetation. Therefore, quantitative analysis can be made statistically from the perspective of the demolition of facilities and the destruction of vegetation.

With regard to the status indicator S, upon completion, this project will create the benefit of ecological restoration and improve tourism resources. However, in the meantime, human activities will also cause adverse effects on trees and vegetation in the park. Therefore, the above indicators can be quantified through the monitoring and management of the smart park after its completion.

As to the influencing factor I, as this project is a public park, influencing factors are mainly economic, environmental, and social benefits. The economic benefit mainly comes from land appreciation. This project mainly aims to restore the ecological protective forest and build a beach park. At present, the ecological protective forest has been built for an area of 22,269 m^2^, and it is estimated that an area of 74,931 m^2^ will still be built later. Therefore, the environmental benefit can be measured by comparing the measured data of the ecological protective forest in the operation period with the current data or be measured in the long run by the weighted quantitative method of water quality, air improvement, and ecological diversity. In terms of social benefits, the main considerations are the ecological restoration of the coastline, landscape repair, improvement of facilities, etc. Since this indicator can hardly be quantified, it is recommended to adopt the common evaluation of tourists, residents, and decision-makers.

For the response factor R, influencing factors mainly include the related construction investment, whether the construction process is managed properly, and whether the corresponding management measures are well configured after the completion of this project. Thus, the quantitative analysis is mainly made from the aspects of capital, equipment, personnel, and management.

Based on the above analysis of the operation period, quantitative research can be conducted on the indicators proposed in this paper in the future from the following aspects, as shown in Table 6.

In conclusion, as restricted by actual conditions, most indicators cannot be quantified to acquire relevant quantitative data at present. Thus, the accurate comprehensive evaluation of the long-term benefits of this project can be carried out only based on the evaluation criteria and corresponding data obtained after the completion of project construction and the operation for 20–30 years. The analysis of relevant indicators in this paper relies on the qualitative analysis of scholars who have participated in similar projects or have certain research experiences in the field of ecological restoration instead of the quantitative process. Moreover, the model-building and analysis process is scientific. Therefore, the results obtained on this basis is effective and credible, which can provide a valuable scientific basis and reference for the stakeholders of similar projects to make decisions.

## 6. Conclusions

In this paper, the DPSIR theory and cloud model are used to construct the comprehensive evaluation model of EIA for ERP in multiple phases, and the impact of positive and negative indexes is comprehensively considered. The main conclusions are as follows:

(1) In this paper, the DPSIR model is used to construct the comprehensive evaluation index system of environmental impact for ERP from the three perspectives of positive indexes, negative indexes, and neutral indexes, including seven positive indexes, four negative indexes, and three neutral indexes. The neutral indexes will show a positive or negative impact due to their implementation. The index system comprehensively considers the positive and negative impact of indexes on the environment. The evaluation model based on the index system can more accurately obtain the actual effect and anticipated effect of ERP.

(2) In this paper, the cloud model and Monte Carlo model are used to analyze and evaluate the multi-phase environmental impact of ERP. It is pointed out that the whole-process benefits of ERP are positive under the impact of negative indexes, but the benefits of ecological restoration in the construction period and short-term operation period are small, and the EIA grade is I (low). However, the EIA grade in the construction period and short-term operation period is III (medium) without considering the negative impact, and the EIA grade in the long-term operation period is IIII (large). Negative indexes will significantly reduce the risk level. Managers should take active response measures to maintain a positive impact on response factors and should set a certain management threshold for negative pressure indexes to avoid damage to the environment at a rate that is faster than the normal restoration speed of the ecosystem. Whether considering negative factors or not, the comprehensive assessment grade of environmental impact for ERP has increased significantly in the long-term operation period. This shows that the scientific effectiveness and technical potential of ERP need to be evaluated within the framework of long-term sustainable development.

Great uncertainty exists in the long-term operation of ERP, so it is necessary to implement iterative management and to follow the cycle management of Plan, Do, Check and Act. In this paper, the treatment of positive and negative indexes is analyzed only in a few phases with relatively long intervals, and the real-time management of positive and negative indexes in the whole process is not achieved. When the management and investment of ERP change, the positive and negative impacts of some indexes, such as management and community participation, will change. In future research, it is necessary to perform the periodic evaluation of indexes at a smaller time scale to improve research methods and to put forward more effective management policies.

## Figures and Tables

**Table 1:** Comprehensive environmental impact evaluation indicators for ecological restoration projects.

	Index	Index Attribute	Index Meaning
D	D1	Positive	Economic development
	D2	Negative	Population size
P	P1	Negative	Construction development intensity
	P2	Negative	Damage intensity of the original environment
	P3	Negative	Pollution intensity during the operation period
S	S1	Positive	The expected improvement of ecological restoration of the project
	S2	Positive	Improvement of tourism resources
	S3	Negative	Pollution caused by human activities during operation
I	I1	Positive	Economic benefits of land appreciation
	I2	Positive	Environmental benefits
	I3	Positive	Social benefits
R	R1	Neutral	Project investment
	R2	Neutral	The degree of control over pollution and damage during construction
	R3	Neutral	Participation and management level during operation

**Table 2:** Standard cloud parameters.

Grade Descriptions	Grade	Interval	Standard Cloud Model Parameters
Small	I	(0,0.2)	(0.1,1/30,0.1)
Little	II	(0.2,0.4)	(0.3,1/30,0.1)
Medium	III	(0.4,0.6)	(0.5,1/30,0.1)
Relatively great	IV	(0.6,0.8)	(0.7,1/30,0.1)
Great	V	(0.8,1.0)	(0.9,1/30,0.1)

**Table 3:** The specific information of experts.

Experts’ Work Units	Number of Experts	Awareness Level of the Project	Notes
China Construction Third Engineering Bureau Co. Ltd.	2	Participation	Constructor
Sanya Yazhou Bay Science and Technology City Administration	2	Participation	Owner
Sanya Institute of Oceanography, Ocean University of China	2	Understanding	Universities engaged in marine ecosystem research, with knowledge of the project
Second Institute of Oceanography, Ministry of Natural Resources	2	Understanding	Research institutions engaged in marine ecosystem research, with knowledge of the project
Hainan Research Institute of Wuhan University of Technology	2	Participation	Subject participant, involved in the preparation of the project construction comprehensive study report

**Table 4:** Index weights and cloud parameters in the construction period.

Indexes	Positive and Negative Weights	*Ex*	*En*	*He*
D1	0.0710	0.3200	0.1354	0.0588
D2	−0.0561	0.2900	0.0677	0.0294
P1	−0.0878	0.7000	0.1003	0.0573
P2	−0.0889	0.6900	0.1153	0.0322
P3	−0.0873	0.1500	0.0877	0.0218
S1	−0.0892	0.5000	0.1253	0.0123
S2	0.0416	0.0700	0.0702	0.0192
S3	0.0286	0.0600	0.0752	0.0277
I1	0.0887	0.2500	0.1003	0.0247
I2	0.0873	0.6500	0.0877	0.0218
I3	0.0561	0.3900	0.0677	0.0294
R1	0.0899	0.5800	0.1253	0.0244
R2	0.0710	0.3200	0.0802	0.0146
R3	0.0565	0.5300	0.1128	0.0387
Comprehensive cloud parameters	-	0.0362	0.0167	0.0044

**Table 5:** Index weight and cloud parameters in the long-term operation period.

Indexes	Positive and Negative Weights	*Ex*	*En*	*He*
D1	0.0659	0.6200	0.1354	0.0358
D2	−0.0823	0.6200	0.1303	0.0185
P1	−0.0153	0.0500	0.0752	0.0256
P2	−0.0802	0.2300	0.0702	0.0192
P3	−0.0690	0.6500	0.1253	0.0201
S1	0.0815	0.7000	0.1003	0.0573
S2	0.0804	0.7100	0.0927	0.0592
S3	0.0815	0.7000	0.1003	0.0573
I1	0.0668	0.6700	0.1203	0.0321
I2	0.0802	0.8300	0.0702	0.0192
I3	0.0672	0.5500	0.1128	0.0325
R1	0.0835	0.5800	0.1253	0.0244
R2	0.0806	0.7600	0.0852	0.0123
R3	0.0657	0.3300	0.0877	0.0303
Comprehensive Cloud Parameters	-	0.3332	0.0394	0.0185

**Table 6:** Research directions for quantification of indexes.

First-Level Indicator	Second-Level Indicator	Acquisition of Quantitative Data
D	Economic development (D1)	GDP of the new district
	Population size (D2)	The ratio of the population of the new district to the current population
P	Construction development intensity (P1)	Number of original facilities demolished; area of the vegetation destroyed by construction
	Damage intensity of the original environment (P2)	The coverage area of the original forest vegetation; frequency of mountain torrents
	Pollution intensity during the operation period (P3)	Changes in air quality; changes in water quality; reduction in vegetation area involved in this project
S	The expected improvement of ecological restoration of the project (S1)	Ecological restoration area of the ecological protective forest; increased categories of birds; improvement of water quality
	Improvement of tourism resources (S2)	Statistics on visitor flow rate in the smart park
	Pollution caused by human activities during operation (S3)	Vegetation reduction area in the operation period
I	Economic benefits of land appreciation (I1)	Value added on land
	Environmental benefits (I2)	Measured data of the ecological protective forest in operation period; the weighting of the water quality, air improvement, and ecological diversity
	Social benefit (I3)	-
R	Project investment (R1)	Comparison between the budget and actual investment in the construction period
	The degree of control of pollution and damage during construction (R2)	Inspection times of the disposal of construction waste, supervision journals of the supervision unit
	Participation and management level during operation (R3)	Qualification of the management unit, investment of the management unit, the configuration of management equipment and personnel

## Data Availability

The case analysis data used to support the findings of this study are available from the corresponding author upon request.
